# Exploring the potential of synthetic and biological fungicides for managing the fungus-farming ambrosia beetle *Xylosandrus compactus*

**DOI:** 10.1371/journal.pone.0329063

**Published:** 2025-07-31

**Authors:** Mariangela Benedetta Costanzo, Alessandro Vitale, Antonio Biondi, Giancarlo Polizzi, Antonio Gugliuzzo

**Affiliations:** Department of Agriculture, Food and Environment, University of Catania, Catania, Italy; University of Carthage, TUNISIA

## Abstract

Little is known about effective control strategies targeting the invasive ambrosia beetle *Xylosandrus compactus*. This fungus-farming beetle is highly dependent on its primary nutritional fungal mutualist *Ambrosiella xylebori*. Traditionally, insect pest control programs target the pest directly. Here, we tested the potential of synthetic and microbial based fungicides to suppress the fungal mutualist, consequently hampering the beetle development. Thiophanate-methyl application to bay laurel (*Laurus nobilis* L.) stem sections proved to be effective in reducing the mutualist fungus occurrence in infested galleries, as well as to reduce the mean *X. compactus* brood size. Thiophanate-methyl and azoxystrobin significantly reduced the mean beetle brood size in extended laboratory conditions. Similarly, these two fungicides were the most effective in reducing the fungal lesion length, both when tested by soil or spray applications. Overall, thiophanate-methyl showed the highest reduction of the *X. compactus* brood size by spray application. No or low impact on *X. compactus* infestations was observed when testing the triazole mefentrifuconazole. Among tested microbial based fungicides, *Trichoderma asperellum* T34 was the only one causing a reduction of the fungal lesion length. To the best of our knowledge, this study provides, for the first time, baseline data on the potential of fungicides for disrupting the mutualistic interaction between *X. compactus* and its primary mutualist *A. xylebori*. These findings will help in developing novel and effective integrated pest management approaches based on the mycobiome alteration and targeting *X. compactus* in its invaded range.

## Introduction

An increasing number of invasive ambrosia beetle species (Coleoptera: Curculionidae: Scolytinae and Platypodinae) is being commonly reported in different non-native regions as a consequence of climate change and global trade [1,2]. More than 50 species of ambrosia beetles are currently known to be established outside their native range, including the xylomycetophagous species belonging to the tribe Xyleborini which can pose a threat to trees growing in forests, nurseries, orchards and urban areas [3,4]. Among them, *Xylosandrus* spp. can become important pests when infesting trees of high economic interest, especially if their associated fungal species act as plant pathogens [5–8].

The most widespread invasive ambrosia beetle species in the *Xylosandrus* genus are *Xylosandrus compactus* (Eichhoff), *Xylosandrus germanus* (Blandford) and *Xylosandrus crassiusculus* (Motschulsky) [9,10]. Native to Asia, they are now widespread, and their association with new and non-coevolved host plants can cause serious damage and economic losses, as has occurred in urban areas and orchards in many regions, including the Mediterranean [11–15].

*Xylosandrus compactus*, commonly known as the black twig borer, is an extremely polyphagous ambrosia beetle [16–17]. It has been found to attack more than 220 plant species belonging to 62 families of trees and shrubs, including agricultural crops [9,16]. *Laurus nobilis* L. and *Ceratonia siliqua* L. have been reported among the most commonly attacked host plants in Southern Europe [13,17–20].

As in the case of other ambrosia beetles*,* controlling *X. compactus* is challenging due to the lack of effective management strategies and because of the cryptic life cycle of the beetle, which occurs almost exclusively within galleries bored in the wood of the host plants [9,16]. Moreover, *X. compactus* larvae do not feed on the wood, but on mutualistic fungi growing into colonized galleries. The fungal spores are transported by the foundresses in dedicated fungus spore–carrying organs called mycetangia [21–22]. *Ambrosiella xylebori* (Brader ex Arx) is the most commonly associated nutritional fungal mutualist of *X. compactus* [9,11,16,23]. However, other fungi have been less frequently isolated from the beetle body or infested galleries, including *Fusarium* spp., *Thyridium* spp., *Geosmithia* spp. and others [6,8,11,24–26].

More recently, a novel phytopathogenic fungal species, *Thyridium lauri* (Voglmayr, D. Aiello & G.R. Leonardi), has been reported in association with *X. compactus* in the Mediterranean [8], and this association seems to be widespread [27]. Furthermore, *Neocosmospora solani* (Martius) L. Lombard & Crous [syn. *Fusarium solani* (Mart.) Sacc.] has also been considered an important symbiont of *X. compactus* in some regions [9,16,28], but its actual role, as well as that of other commonly associated fungal species, in the bio-ecology of *X. compactus* needs further investigation [25,29].

The life cycle and host selection behavior of *X. compactus* may vary depending on the climatic conditions and host range availability in the specific invaded region, thus affecting the potential effectiveness of pest management protocols. For example, in the southern Mediterranean environment, *X. compactus* has been reported to attack twigs, branches and trunks of *C. siliqua* completing up to five generations per year, being active from April to late autumn [11,19]. By contrast, in Uganda the pest can be active throughout the year infesting coffee twigs [30]. These studies suggest that monitoring and pruning should be carried out on different infested tissues and at different time intervals depending on locations [9,11,19,30]. Furthermore, what makes *X. compactus* a threat for economically important cultivated trees is its ability to infest not only stressed plants, but also apparently healthy plants [16,19,20].

In this context, research efforts are aimed at finding effective management approaches targeting both ambrosia beetles and their associated fungi, while minimizing environmental risks and human health [9,31]. As part of Integrated Pest Management (IPM) programs, this objective can be achieved by combining different approaches, including biological, cultural, semiochemical, mechanical, physical and other tools [9,32]. Because ambrosia beetles are strongly dependent on mutualistic fungi as unique source of food, sustainable and potentially effective management strategies targeting the alteration of their microbial community can represent suitable approaches for managing their infestations [25,33–36]. For example, promising results have been obtained when evaluating biocontrol agents, i.e., *Trichoderma* spp. and *Bacillus* spp., to suppress microbial mutualism between *X. compactus* and its main nutritional fungal mutualist *A. xylebori* [25]. Other studies also showed that the use of synthetic fungicides may be suitable for managing infestations by some ambrosia beetle species [3,31,33,35–37].

However, the potential of synthetic fungicides for managing infestations by *X. compactus* still needs to be studied. Previous investigations have tested the potential of fungicides to inhibit the growth of *X. compactus* associated fungi only *in vitro.* In detail, chlorothalonil, dimethomorph + mancozeb, tebuconazole, and propiconazole effectively inhibited growth of *X. compactus* fungal associates under laboratory conditions [38,39]. No previous study has specifically focused on testing the application of fungicides to susceptible plants to manage *X. compactus* infestations.

Thus, the present study aims to evaluate the potential of applications of commercially available fungicides for suppressing mutualistic interactions between the invasive ambrosia beetle *X. compactus* and its main nutritional fungal symbiont *A. xylebori*. Specific bioassays were conducted (i) to evaluate the potential repellent effect of systemic synthetic fungicides towards *X. compactus* under laboratory conditions, and (ii) to assess the potential impact of both selected synthetic and microbial-based fungicides to control this invasive ambrosia beetle by suppressing its main nutritional mutualist under laboratory and extended laboratory conditions.

## Materials and methods

### Beetle laboratory rearing

The *X. compactus* laboratory colony was established as described by Gugliuzzo et al. (2022) [25] and maintained for several generations according to Gugliuzzo et al. (2023) [40]. Briefly, the ambrosia beetle was reared on healthy *L. nobilis* stem sections with a diameter of 7–15 mm, collected at the campus of the University of Catania (Catania, Italy). After field selection, stems were cut into 12–15 cm long sections and the leaves removed. Each stem section was then covered at both ends with Parafilm^®^ strips to reduce drying. These were then immersed in a 10% EtOH solution (in sterile distilled water – SDW) for two hours to make them more attractive to beetle females [25,41,42]. After immersion, bay laurel stems sections were left to dry on sterile filter paper for 30 min under a laminar flow hood. These were then transferred into individual 25 × 250 mm culture glass tubes, where six to eight *X. compactus* female adults emerged from the previous rearing were released. Tubes were sealed with wet cellulose acetate caps and moistened with distilled water, whenever needed. The tubes were kept in the dark at 25 ± 2°C and 60 ± 10% RH. Thirty-five ± two days after releasing beetle foundresses, new emerging adult offspring were collected for conducting experimental bioassays or for maintaining the rearing colony.

### Tested synthetic and microbial fungicides

Three commercial synthetic fungicides were tested in both laboratory and extended laboratory conditions; while three biological fungicides previously tested under laboratory conditions in Gugliuzzo et al. (2022) [25] were tested in extended laboratory conditions only. Details concerning each of the tested fungicides are provided in Table 1. To the best of our knowledge, there is no commercial fungicide specifically authorized in Europe for managing *X. compactus* infestation by targeting its fungal mutualist. In the present study, synthetic fungicides (i.e., azoxystrobin, mefentrifluconazole and thiophanate-methyl) were selected for their well-known systemic properties and different modes of actions.

**Table 1:** Synthetic and microbial-based fungicides tested against *Xylosandrus compactus* and its fungal mutualist *Ambrosiella xylebori.*

Active ingredient (a.i.)	Class	Concentration a. i.	Trade name and manufacturer
Azoxystrobin	QoI-fungicides (Quinone outside Inhibitors)	250 g/L	Ortiva®, Syngenta
Mefentrifluconazole	DMI-fungicides (DeMethylation Inhibitors)	75 g/L	Revysion®, BASF
Thiophanate-Methyl	MBC fungicides (Methyl Benzimidazole Carbamates)	500 g/L	Enovit metil® FL, Sipcam
*Trichoderma asperellum* ICC 012 + *Trichoderma gamsii* ICC 080	Microbial fungicide	3 × 10^7^ CFU/g	Remedier®, Gowan
*Trichoderma asperellum* T34	Microbial fungicide	1 × 10^9^ CFU/g	T-34 Biocontrol®, Biolchim
*Bacillus amyloliquefaciens D747*	Microbial fungicide	1 × 10^10^ CFU/g	Amylo-X LC®, Biogard

In particular, azoxystrobin was selected for its broad spectrum and systemic and translaminar activity, it is also authorized in Europe and United States for use on ornamental plants. Previous studies revealed a reduction in ambrosia beetle (i.e., *X. germanus*) infestations on treated trees exposed to flooding in USA [31,43]. Mefentrifluconazole was selected because it is an innovative triazole with translaminar and systemic activity that can be active over a wide temperature range on *Fusarium* spp., *Colletotrichum* sp. and other phytopathogens [44–48]. In addition, it is rapidly absorbed by the vegetation which can improve its effectiveness [44,49,50]. Thiophanate-methyl was selected for its systemic activity, broad spectrum of action and well-known ability to control tracheomycotic fungi on ornamental plants [51,52].

Biofungicides based on specific strains of *Trichoderma* mycoparasitic fungi and the antagonistic bacteria *Bacillus amyloliquefaciens* strain D747, were selected as they are commonly used as Biological Control Agents (BCAs) against plant pathogenic fungi in several contexts [53–56]. Moreover, some of these BCAs previously showed the ability to suppress the growth of *A. xylebori*, the main mutualist of *X. compactus*, and consequently hamper the production and development of its progeny on carob twigs in the laboratory [25].

One of the mechanisms by which these microbials indirectly perform their biocontrol role is by acting as elicitors of Induced Systemic Resistance (ISR), mediated by signaling phytohormones, like jasmonic acid (JA) and salicylic acid (AC). These hormones regulate the plant’s defensive responses, leading it to produce defense proteins and phytoalexins [57,58]. This mechanism has been identified in a variety of soil or air-borne pathogen/plant systems, but it is still poorly investigated for fungi associated with wood-boring beetles [57,59–62].

### Choice bioassays in the laboratory

Dual choice bioassays were used to assess the potential effect of synthetic fungicide application to bay laurel stems on the behavior of *X. compactus*. In particular, the beetle preference between stem sections treated with fungicides and untreated control stem sections was evaluated within specific experimental arenas (made by plastic boxes, 175 × 120 × 70 mm) where adult beetle females were left free to start the infestation (bore the entry hole) in one of the two stems [40]. Each box was provided with a window (135 × 80 mm) covered with a fine mesh net (0.25 × 0.25 mm) to facilitate ventilation.

As done for the beetle rearing, healthy bay laurel stems with a diameter of 7–15 mm and about 12 cm long were collected at the campus of the University of Catania (Catania, Italy), covered at both ends with Parafilm^®^ strips and soaked in a 10% EtOH solution (in SDW) for two hours before being dried for 30 min. Then, stem sections were dipped for 10 s into the different fungicide solutions prepared following the highest label Field Rates (FR), consisting in 1 mL L^–1^ for azoxystrobin, 2 mL L^–1^ for mefentrifluconazole and 1.5 mL L^–1^ for thiophanate-methyl, or into SDW (untreated control). After drying for 30 min, a fungicide treated stem section and a control stem section were placed at the opposite sides of each plastic box (replicate) and five beetle females were released in the center of the arena. For each treatment (fungicide *vs* control), there were 10 replicates (boxes), and a total of 50 tested females. Boxes were kept in the darkness at 25 ± 1 C° and 60 ± 10% RH.

The *X. compactus* preference was assessed 24 h, 48 h and 72 h after the beetle releases by counting the number of individuals making entry holes in each of the two stem sections (treated *vs* untreated). After 72 h, beetle females that were not found boring entry holes in one of the two stem sections were considered as having made no choice and excluded from data analysis.

### Infestation success bioassays in the laboratory

No-choice bioassays were used to evaluate the potential of selected synthetic fungicides to affect *X. compactus* infestation success in treated host wood. Stem sections were collected, prepared and treated with synthetic fungicides with the same methodology as the previous section. The only difference was that each individual treated stem was transferred into a sterile glass tube (25 × 250 mm culture tubes), where five *X. compactus* foundresses from the laboratory rearing were released. Eight replicates (glass tubes), and a total of 40 beetle females were tested for each fungicide and for the untreated control. Each tube was then closed with a wet cellulose acetate plug and kept in darkness at 25 ± 1 C° and 60 ± 10% RH for 21 days.

The number of *X. compactus* foundresses that survived the treatment and started to bore entry holes was recorded 24 h, 48 h and 72 h after their release. After 21 days, stem sections were dissected and single galleries were observed under a stereomicroscope to evaluate the number of foundresses i) establishing a gallery, ii) cultivating the fungal mutualist, and iii) producing progeny. Moreover, the brood size, as the total number of produced offspring per foundress, was recorded.

### Extended laboratory bioassays

All the synthetic fungicides reported in Table 1 were tested in extended laboratory bioassay with two different application methods, i.e., foliar and soil application. Foliar application consisted of spraying leaves, branches and the main stem of bay laurel plants with the highest label field rates, i.e., 0.25 g L^-1^ for azoxystrobin, 0.15 g L^-1^ for mefentrifluconazole and, 0.75 g L^-1^ for thiophanate-methyl. In particular, potted bay laurel plants (pot diameter = 18 cm) were grouped in randomized blocks before being stressed by flooding and then sprayed until runoff with fungicides or water (control). Bay laurel plants were approximately 2–3-year-old and 1.20–1.40 m tall. Plants were physiologically stressed starting from 4 days before treatment by imposing flooding through a pot-in-pot system [63]. This stress condition simulates suitable field conditions anticipating ambrosia beetle attacks to flood-stress susceptible plants [31,41,64,65]. For this purpose, pots with a diameter of 24 cm were lined with waterproof plastic bags. Potted bay laurel plants were placed inside the lined plot and irrigated until there was standing water around the plant base. After four days of flooding stress, each plant was sprayed with 100 ml of synthetic fungicide solution from a distance of 0.3 m using a hand sprayer (Dea 2000 Volpi^®^, Italy) and left to dry. Untreated control plants were sprayed only with water (S1 Fig).

The stems of treated bay laurel plants were exposed to dispersing coetaneous beetle females at three different time intervals after treatment (1 DAT, 1 day after treatment; 3 DAT, 3 days after treatment; 7 DAT, 7 days after treatment). In particular, for each treatment/exposure time combination, five experimental chambers, consisting of 1.5 ml Eppendorf tubes (Aptaca, Italy) were attached along the main stem of each plant starting from 8 cm above the soil surface and then 15 cm apart from each other, following the methodology provided by Costanzo et al. (2025) [66]. A single *X. compactus* dispersing female from the laboratory rearing was released inside each chamber. A total of 5 plants and 25 beetle females for each fungicide/time of exposure combination were tested, as for the control, with a total of 300 tested individuals (S1 Fig).

A similar experimental design, with a few adaptations, was used to evaluate the potential of both synthetic and microbial based fungicides listed in Table 1 via soil application on potted bay laurel plants. To this aim, each bay laurel potted plant was irrigated with 200 mL of fungicide solutions. Tested synthetic fungicide formulations were based on an active ingredient amount (diluted in water) resulting from the suggested label rate per hectare and adapted depending on the number of plants occurring per square meter, i.e., of 0.075 g plant^-1^ for azoxystrobin, 0.045 g plant^-1^ for mefentrifuconazole, and 0.225 g plant^-1^ for thiophanate-methyl. Biofungicide solutions were prepared following previous studies and suggested label field rates, consisting of 5 mL L^-1^ (commercial formulation in water) for *B. amyloliquefaciens* D747, 5 g L^-1^ for *Trichoderma asperellum* T34 and 5 g L^-1^ for *T. asperellum* strain ICC 012 + *T. gamsii* strain ICC 080. Three time-intervals after treatment (1 DAT; 3 DAT; 7 DAT) were tested for the synthetic fungicides, while only one-time interval after treatment (7 DAT) was tested for the biological ones, considering the time needed to these microbials to start growing in the inoculated soil. A total of 25 beetle females for each fungicide/time of exposure combination was tested, as for the control, with a total of 375 tested individuals.

Plants were first checked 24 h and 48 h after beetle female releases for evidence of frass production and gallery initiation as confirmation that the beetles entered the wood or to detect if boring activity did not occur. The potential effect of the commercial fungicides to manage mutualistic association between *X. compactus* and its main fungal symbiont was evaluated two weeks after insect release. To this aim, each plant was cut into portions, each including a maternal gallery belonging to a single beetle foundress. As in the previous section, each stem section was dissected and observed under a stereomicroscope to record evidence of tunneling (gallery establishment), fungal mutualist growth, progeny occurrence, and brood size. The internal fungal lesion length of woody tissues around each infested gallery, for all foundresses belonging to different treatments, was also measured.

### Data analysis

The data belonging to the choice laboratory bioassay were analysed by means of the Chi-square (χ²) test to compare whether the beetle choice between fungicide treated and untreated bay laurel stem sections was significantly different from a 50:50 distribution. Raw data from laboratory and extended laboratory bioassays were first tested for normality and homogeneity of variance through Kolmogorov–Smirnov and Shapiro–Wilk tests before further analysis were conducted. For infestation success laboratory bioassays, the mean percentages of alive beetles, boring beetles, beetles producing galleries (evidence of tunneling), cultivating fungal mutualist, producing progeny and the brood size (mean number of offspring produced by foundresses) were calculated. For extended laboratory bioassays, the mean percentages of beetles producing galleries, cultivating fungal mutualist, and producing progeny, as well as the brood size and the internal vascular lesion caused by the fungal symbiont were calculated. The brood size was calculated considering only those beetle females that survived the fungicide treatments and untreated control groups. Because the obtained data did not fulfil the assumptions for analysis of variance (ANOVA), the non-parametric Kruskal-Wallis test followed by Dunn’s post hoc test (*p* < 0.05) were used for multiple comparisons of means among treatments. Statistical analyses were carried out using IBM^®^ SPSS^®^ Statistics, Version 23.0.0.0 (IBM Corp., Armonk, NYUSA), R software v3.3.2 and RStudio v.2023.12.0−369.

## Results

### Choice bioassays in the laboratory

*Xylosandrus compactus* females showed no significant preference when allowed to choose between fungicide-treated and untreated bay laurel stem sections. In particular, the proportion of individuals colonizing treated *vs* untreated twigs was not significantly different from a 50:50 type distribution for all tested fungicides at 24 h, 48 h and 72 h after their release (24h: azoxystrobin: χ² = 0.419; *p* = 0.517; thiophanate-methyl: χ² = 0.383; *p* = 0.536; mefentrifluconazole: χ² = 1.500; *p* = 0.221; 48h and 72h: azoxystrobin: χ² = 0.727; *p* = 0.394; thiophanate-methyl: χ² = 0.167; *p* = 0.683; mefentrifluconazole: χ² = 1.500; *p* = 0.221).

### Infestation success bioassays in the laboratory

The number of alive beetle individuals was not significantly affected by exposure to fungicide-treated stems at 24 h, 48 h and 72 h after their release. Survival rates were 100% for the control group and ranged from 90.24% to 97.50% for the treated ones. Similarly, there was no significant difference in boring activity between females exposed to treated or untreated twigs, with the percentages of individuals boring into the stem sections ranging from 75.61 ± 6.79% to 92.68 ± 4.12% for the control group and from 67.50 ± 7.50% to 92.50 ± 4.22% for the tested synthetic fungicides (Table 2).

**Table 2:** Impact of synthetic fungicides on *Xylosandrus compactus* survival and boring activity (mean percentage ± SE) after 24 h, 48 h and 72 h of exposure during infestation success laboratory bioassays.

Treatment	% alive beetles (mean ± SE)			% of boring beetles (mean ± SE)		
	24h	48h	72h	24h	48h	72h
Ctrl	100.00^ns^	100.00^ns^	100.00^ns^	75.61 ± 6.79^ns^	90.24 ± 4.69^ns^	92.68 ± 4.12^ns^
Azoxystrobin	97.50 ± 2.50^ns^	92.59 ± 4.22^ns^	95.00 ± 3.49^ns^	87.50 ± 5.30^ns^	90.00 ± 4.80^ns^	92.50 ± 4.22^ns^
Mefentrifuconazole	95.00 ± 3.45^ns^	97.50 ± 2.50^ns^	95.00 ± 3.45^ns^	67.50 ± 7.50^ns^	85.00 ± 5.72^ns^	90.00 ± 4.80^ns^
Thiophanate-Methyl	92.60 ± 4.12^ns^	90.24 ± 4.69^ns^	95.12 ± 3.41^ns^	73.17 ± 7.01^ns^	82.93 ± 5.95^ns^	85.37 ± 5.59^ns^
	*H* = 0.364 *p* = 0.333	*H* = 0.731 *p* = 0.158	*H* = 0.224 *p* = 0.552	*H* = 2.538 *p* = 0.201	*H* = 0.486 *p* = 0.696	*H* = 0.424 *p* = 0.661

Within each column, means (± SE) with ns are not significantly different according to Kruskal-Wallis H test followed by Dunn’s post hoc test for multiple comparison at *p* < 0.05.

Gallery establishment, mutualist growth, offspring production and brood size were significantly affected only by thiophanate-methyl, while mefentrifuconazole and azoxystrobin showed a trend of reduction in beetle brood size, although non-significant, compared to the control 21 days after females releases (Table 3).

**Table 3:** Impact of synthetic fungicides on *Xylosandrus compactus* gallery establishment and brood production during infestation success laboratory bioassays. The percentages of beetle females producing galleries, cultivating mutualist and producing progeny (mean percentage ± SE) 21 days after the exposure are reported. Brood size is calculated as the mean number (± SE) of offspring produced by foundresses per treatment.

Treatment	% with gallery (mean ± SE)	% with mutualist (mean ± SE)	% with progeny (mean ± SE)	Brood size (mean ± SE)
Ctrl	60.53 ± 8.04^a^	60.53 ± 8.04^a^	60.53 ± 8.04^a^	26.09 ± 3.21^a^
Azoxystrobin	55.26 ± 8.17^a^	56.76 ± 8.26^a^	54.05 ± 8.31^a^	16.30 ± 3.59^ab^
Mefentrifuconazole	68.42 ± 7.64^a^	68.42 ± 7.64^a^	68.42 ± 7.64^a^	17.62 ± 1.85^ab^
Thiophanate-Methyl	26.32 ± 7.24^b^	21.05 ± 6.70^b^	21.05 ± 6.70^b^	13.90 ± 1.63^b^
	*H* = 11.450 *p* = 0.001	*H* = 14.820 *p* < 0.001	*H* = 14.650 *p* < 0.001	*H* = 8.683 *p* = 0.033

Within each column, means (± SE) with different letters are significantly different according to Kruskal-Wallis *H* test followed by Dunn’s post hoc test for multiple comparison at *p* < 0.05.

### Extended laboratory bioassays

#### Effect of spray applications of synthetic fungicides.

Synthetic fungicide spray application did not significantly affect the percentage of beetles establishing gallery and cultivating mutualist. On the other hand, there was a significant difference in the progeny occurrence between mefentrifuconazole and thiophanate-methyl when beetles were released at 1 DAT (Table 4). Moreover, decreasing trends in mutualist and progeny occurrence, although not significant, were observed for thiophanate-methyl treated plants when beetles were released at 7 DAT (Table 4).

**Table 4:** Impact of synthetic fungicides applied by spray on *Xylosandrus compactus* gallery establishment, mutualist growth and progeny occurrence (mean percentage ± SE) 14 days after beetle releases on potted bay laurel plants.

Treatment	% with gallery (mean ± SE)			% with mutualist (mean ± SE)			% with progeny (mean ± SE)		
	1 DAT	3 DAT	7 DAT	1 DAT	3 DAT	7 DAT	1 DAT	3 DAT	7 DAT
Ctrl	61.54 ± 9.73^ns^	60.00 ± 10.00^ns^	60.00 ± 10.00^ns^	60.00 ± 10.00^ns^	60.00 ± 10.00^ns^	60.00 ± 10.00^ns^	60.00 ± 10.00^ab^	60.00 ± 10.00^ns^	60.00 ± 10.00^ns^
Azoxystrobin	69.23 ± 9.23^ns^	76.00 ± 8.72^ns^	64.00 ± 9.80^ns^	60.00 ± 10.00^ns^	72.00 ± 9.17^ns^	60.00 ± 10.00^ns^	60.00 ± 10.00^ab^	68.00 ± 9.52^ns^	52.00 ± 10.20^ns^
Mefentrifuconazole	76.00 ± 8.72^ns^	68.00 ± 9.52^ns^	68.00 ± 9.52^ns^	76.00 ± 8.72^ns^	64.00 ± 9.80^ns^	68.00 ± 9.52 ^ns^	76.00 ± 8.72^a^	64.00 ± 9.80^ns^	68.00 ± 9.52^ns^
Thiophanate-Methyl	61.54 ± 9.73^ns^	68.00 ± 9.52^ns^	72.00 ± 9.17^ns^	52.00 ± 10.20^ns^	64.00 ± 9.80^ns^	48.00 ± 10.20^ns^	36.00 ± 9.80^b^	56.00 ± 10.13^ns^	40.00 ± 10.00^ns^
	*H* = 1.101 *p* = 0.646	*H* = 0.950 *p* = 0.692	*H* = 0.594 *p* = 0.829	*H* = 2.257 *p* = 0.362	*H* = 0.564 *p* = 0.843	*H* = 1.515 *p* = 0.554	*H* = 6.163 *p* = 0.037	*H* = 0.594 *p* = 0.839	*H* = 3.178 *p* = 0.232

Each parameter was calculated at 3 different exposure time intervals, i.e., 1 DAT (1 day after treatment), 3 DAT (3 days after treatment) and 7 DAT (7 days after treatment). Within each column, means (± SE) with ns are not significantly different according to Kruskal-Wallis *H* test followed by Dunn’s post hoc test for multiple comparison at *p* < 0.05.

**Fig 1:**
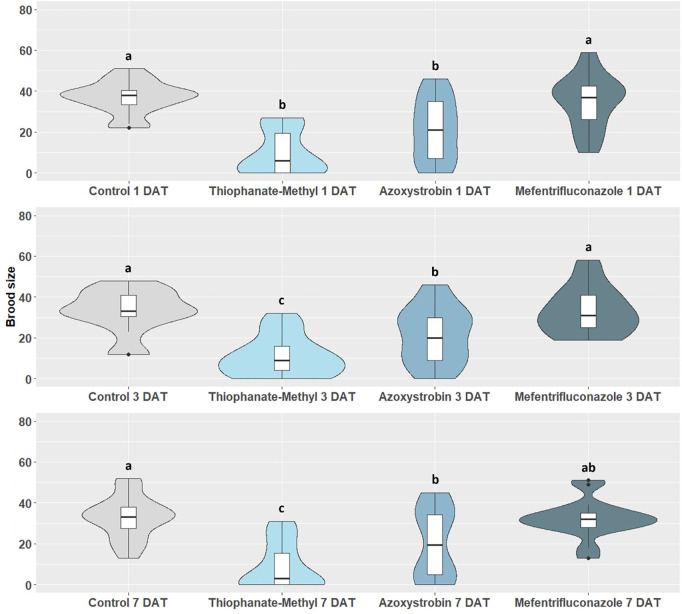
Impact of synthetic fungicide spray applications on *X. compactus* brood size 14 days after beetle releases on potted bay laurel plants. Brood size is calculated as the mean number (± SE) of offspring produced by foundresses at 3 different exposure time intervals, i.e., 1 DAT (1 day after treatment), 3 DAT (3 days after treatment) and 7 DAT (7 days after treatment). Means (± SE) with different letters are significantly different according to Kruskal-Wallis *H* test followed by Dunn’s post hoc test for multiple comparison at *p* < 0.05.

The impact of synthetic fungicide spray application on *X. compactus* brood size is shown in Fig 1. In particular, the mean brood size was significantly affected by thiophanate-methyl and azoxystrobin when compared to the control (1 DAT: *H* = 28.260; *p* < 0.001; 3 DAT: *H* = 26.930; *p* < 0.001; 7 DAT: *H* = 25.580; *p* < 0.001), with thiophanate-methyl causing the largest progeny reduction. The mean brood size produced by *X. compactus* females varied from 9.80 ± 2.79 for thiophanate-methyl to 37.13 ± 2.03 for the control at 1 DAT, from 10.94 ± 2.41 for thiophanate-methyl to 33.24 ± 2.80 for mefentrifuconazole 3 DAT, and from 8.65 ± 2.83 for thiophanate-methyl to 32.93 ± 2.73 for the control at 7 DAT.

#### Effect of soil applications of synthetic and microbial-based fungicides.

None of the tested synthetic and microbial fungicides when applied to the substrate showed a significant impact on gallery production by beetle females, growth of mutualist and progeny occurrence, compared to the control (Tables 5 and 6). However, there were decreasing trends, although not significant, concerning the percentages of beetles boring galleries when *X. compactus* females were released 1 DAT for azoxystrobin, the percentage of galleries with mutualistic growth when beetles were released 1, 3 and 7 DAT respectively for azoxystrobin, thiophanate-methyl and mefentrifuconazole, and in progeny occurrence when beetles were released 3 and 7 DAT for mefentrifuconazole and thiophanate-methyl or *T. asperellum* T34, respectively (Tables 5 and 6).

**Table 5:** Impact of synthetic fungicides applied to the soil on *Xylosandrus compactus* gallery establishment, mutualist growth and progeny occurrence (mean percentage ± SE) 14 days after beetle releases on potted bay laurel plants.

Treatment	% with gallery (mean ± SE)			% with mutualist (mean ± SE)			% with progeny (mean ± SE)		
	1 DAT	3 DAT	7 DAT	1 DAT	3 DAT	7 DAT	1 DAT	3 DAT	7 DAT
Ctrl	61.54 ± 9.73^ab^	76.92 ± 8.43^ns^	72.00 ± 9.17^ns^	61.54 ± 9.73^ns^	73.08 ± 8.87^ns^	68.00 ± 9.52^ns^	46.15 ± 9.97^ns^	68.00 ± 9.34^ns^	64.00 ± 9.80^ns^
Azoxystrobin	46.15 ± 9.97^a^	74.07 ± 8.59^ns^	80.77 ± 7.88^ns^	38.46 ± 9.73^ns^	70.37 ± 8.96^ns^	73.08 ± 8.87^ns^	34.62 ± 9.51^ns^	66.67 ± 9.25^ns^	61.54 ± 9.73^ns^
Mefentrifuconazole	80.77 ± 7.88^b^	80.77 ± 7.88^ns^	69.23 ± 9.23^ns^	73.08 ± 8.87^ns^	69.23 ± 9.23^ns^	50.00 ± 10.00^ns^	61.54 ± 9.73^ns^	57.69 ± 9.88^ns^	46.15 ± 9.97^ns^
Thiophanate-Methyl	61.54 ± 9.73^ab^	51.85 ± 9.80^ns^	76.00 ± 8.72^ns^	57.69 ± 9.88^ns^	51.85 ± 9.80^ns^	72.00 ± 9.17^ns^	46.15 ± 9.97^ns^	51.85 ± 9.80^ns^	48.00 ± 10.20^ns^
	*H* = 5.501 *p* = 0.048	*H* = 4.539 *p* = 0.059	*H* = 0.581 *p* = 0.796	*H* = 5.451 *p* = 0.058	*H* = 2.400 *p *= 0.308	*H* = 2.658 *p* = 0.269	*H* = 3.311 *p* = 0.219	*H* = 1.085 *p* = 0.677	*H* = 1.900 *p* = 0.465

Each parameter was calculated at three different exposure time intervals, i.e., 1 DAT (1 day after treatment), 3 DAT (3 days after treatment) and 7 DAT (7 days after treatment). Within a column, means (± SE) with different letters are significantly different according to Kruskal-Wallis *H* test followed by Dunn’s post hoc test for multiple comparison at *p* < 0.05. ns = not significant.

**Table 6:** Impact of microbial-based fungicides applied to the soil on *Xylosandrus compactus* gallery establishment, mutualist growth and progeny occurrence (mean percentage ± SE) 14 days after beetle releases on potted bay laurel plants.

Treatment	% with gallery (mean ± SE)	% with mutualist (mean ± SE)	% with progeny (mean ± SE)
	7 DAT	7 DAT	7 DAT
Ctrl	72.00 ± 9.17^ns^	68.00 ± 9.52^ns^	64.00 ± 9.80^ns^
*Trichoderma asperellum* ICC 012 + *Trichoderma gamsii* ICC 080	70.37 ± 8.96^ns^	70.37 ± 8.96^ns^	59.26 ± 9.64^ns^
*Trichoderma asperellum* T34	75.00 ± 9.03^ns^	62.50 ± 10.09^ns^	54.17 ± 10.39^ns^
*Bacillus amyloliquefaciens* D747	76.92 ± 8.43^ns^	76.92 ± 8.43^ns^	69.23 ± 9.23^ns^
	*H* = 0.201 *p* = 0.951	*H* = 0.797 *p* = 0.739	*H* = 0.929 *p* = 0.726

Within a column, means (± SE) with ns are not significantly different according to Kruskal-Wallis *H* test followed by Dunn’s post hoc test for multiple comparison at p < 0.05. 7 DAT = 7 days after treatment.

**Fig 2:**
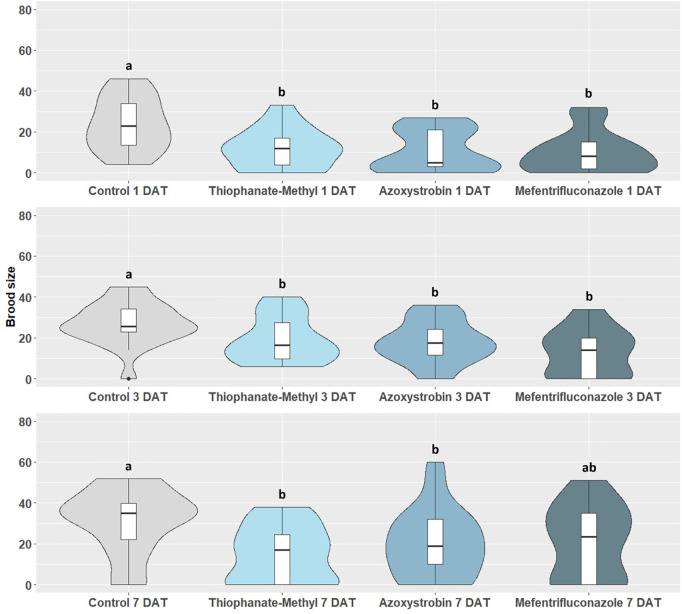
Impact of synthetic fungicides applied to the soil on *X. compactus* brood size 14 days after beetle releases on potted bay laurel plants. Brood size is calculated as the mean number (± SE) of offspring produced by foundresses at 3 different exposure time intervals, i.e., 1 DAT (1 day after treatment), 3 DAT (3 days after treatment) and 7 DAT (7 days after treatment). Means (± SE) with different letters are significantly different according to Kruskal-Wallis *H* test followed by Dunn’s post hoc test for multiple comparison at *p* < 0.05.

The mean *X. compactus* brood size was significantly affected by synthetic fungicides application to the soil of tested bay laurel plants (1 DAT: *H* = 8.025; *p* = 0.044; 3 DAT: *H* = 14.470; *p* = 0.002; 7 DAT: *H* = 10.510; *p* = 0.013). However, no significant difference was found between the three tested active ingredients, which caused a similar trend of offspring reduction (Fig 2). In particular, the *X. compactus* brood size varied from 10.10 ± 2.19 for mefentrifuconazole to 22.53 ± 3.70 for the control group at 1 DAT, from 13.29 ± 2.45 for mefentrifuconazole to 26.95 ± 2.33 for the control group at 3 DAT, and from 14.84 ± 3.16 for thiophanate-methyl to 31.44 ± 3.69 for the control group when *X. compactus* females were released on bay laurel plants 7 DAT (Fig 2).

**Fig 3:**
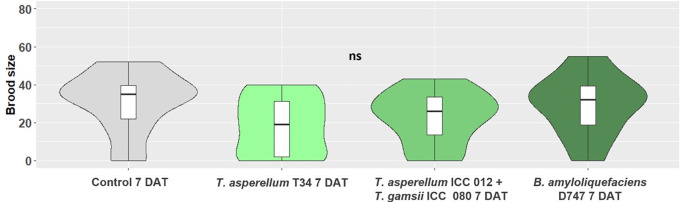
Impact of microbial-based fungicides applied to the soil on *X. compactus* brood size 14 days after beetle releases on potted bay laurel plants. Brood size is calculated as the mean number (± SE) of offspring produced by foundresses at 7 DAT (7 days after treatment). ns indicates no significant differences according to Kruskal-Wallis *H* test at *p* < 0.05.

There was no significant difference in the brood size produced by the beetle foundresses on bay laurel plants previously treated with microbial-based fungicides (Fig 3). However, a trend of reduction was observed when *X. compactus* females were released on plants treated with *T. asperellum* strain T34 (brood size = 18.67 ± 3.59).

#### Effect of soil and spray applications of fungicides on fungal mutualist lesion length.

A significant reduction on the length of vascular lesion was observed only when testing azoxystrobin and thiophanate-methyl both by spray (1 DAT) and soil (7 DAT) applications (Table 7). Among the microbial-based fungicides, only *T. asperellum* T34 significantly reduced the fungal lesion length (29.47 ± 4.99 mm) compared to the control (60.65 ± 6.38 mm) (*H* = 15.320; *p* = 0.001). *Trichoderma asperellum* strain ICC 012 + *T. gamsii* strain ICC 080 (lesion length: 44.11 ± 3.23 mm) and *B. amyloliquefaciens* strain D747 (lesion length: 50.40 ± 5.19 mm) showed a trend of reduction, although not significant, compared to the control.

**Table 7:** Effect of synthetic fungicides application to the soil on vascular lesion length (mm) (mean ± SE) caused by the fungal symbiont 14 days after beetle releases on bay laurel plants at 3 different exposure time intervals, i.e., 1 DAT (1 day after treatment), 3 DAT (3 days after treatment) and 7 DAT (7 days after treatment).

Treatment	Soil application – Lesion length (mm) (mean ± SE)			Spray application – Lesion length (mm) (mean ± SE)		
	1 DAT	3 DAT	7 DAT	1 DAT	3 DAT	7 DAT
Ctrl	50.13 ± 7.09^ns^	43.63 ± 5.10^ns^	60.65 ± 6.38^a^	61.20 ± 4.43^a^	63.67 ± 3.79^ns^	55.27 ± 5.18^ns^
Azoxystrobin	35.36 ± 4.93^ns^	36.79 ± 3.67^ns^	40.42 ± 4.39^b^	40.73 ± 4.82^bc^	63.28 ± 5.88^ns^	40.07 ± 5.84^ns^
Mefentrifuconazole	47.52 ± 5.58^ns^	33.67 ± 3.26^ns^	53.27 ± 5.72^ab^	55.68 ± 4.39^ab^	71.69 ± 4.74^ns^	46.29 ± 6.36^ns^
Thiophanate-Methyl	42.27 ± 7.08^ns^	34.64 ± 3.10^ns^	36.78 ± 4.04^b^	32.47 ± 4.79^c^	55.88 ± 5.96^ns^	43.75 ± 5.36^ns^
	*H* = 2.727 *p* = 0.435	*H* = 1.258 *p* = 0.738	*H* = 9.645 *p* = 0.021	*H* = 16.550 *p* = 0.001	*H* = 3.472 *p* = 0.324	*H* = 5.525 *p* = 0.136

Within a column, means (± SE) with different letters are significantly different according to Kruskal-Wallis *H* test followed by Dunn’s post hoc test for multiple comparison at *p* < 0.05. ns = not significant.

## Discussion

Conventional control strategies of beetle pests and related plant diseases are mostly based on chemical fungicides and insecticides [33,67,68]. However, conventional approaches targeting invasive ambrosia beetles are often ineffective because of the pest broad host range, their rapid spread, and cryptic life cycle inside the host wood [3,9,31]. As a consequence, the development of effective, alternative and innovative management strategies directly targeting the beetles and/or their fungal associates is currently challenging [9].

To the best of our knowledge, the present study is the first one focusing on the use of fungicide applications as a management approach for *X. compactus*. Because ambrosia beetles live in nutritional symbiosis with ambrosia fungi, chemical and microbial based fungicides can have the potential to act as a valid alternative to hamper their infestations [25,33,35–37,42,67]. We demonstrated how soil or spray application of specific fungicides can affect the growth of the fungal mutualist and consequentially impact the *X. compactus* progeny.

Laboratory bioassays showed that none of the tested synthetic fungicides had an impact on *X. compactus* survival and boring activity after exposure to treated bay laurel stems. However, the proportion of galleries with mutualistic fungal growth and offspring occurrence were significantly reduced by the tested thiophanate-methyl application (Tables 2 and 3). The mean brood size was also significantly reduced when testing the same fungicide under laboratory conditions. Moreover, thiophanate-methyl was the most effective active ingredient in reducing the *X. compactus* brood size, especially by spray application, and in reducing the fungal lesion length in extended laboratory bioassays (Figs 1 and 2, Table 7). Altogether, these findings suggest that thiophanate-methyl can be considered an active ingredient interfering with the mutualist fungal growth and consequently affecting progeny production. Thiophanate-methyl belongs to Methyl Benzimidazole Carbamate (MBC) fungicides and it is still present on the market in some regions where *X. compactus* is present and considered a pest of economic interest, including USA, and countries of Africa and Asia [69–71]. Based on our initial results, other benzimidazole compounds should be investigated as potential candidates to manage *X. compactus* infestations.

Previous studies evaluated the potential of azoxystrobin or other Quinone outside Inhibitor (QoI) fungicides to reduce ambrosia beetle attacks, including other *Xylosandrus* spp. QoI fungicides can reduce pest and pathogen damages by promoting the host immune response [3,31,72,73]. Moreover, the “stress mitigating” properties of QoIs could indirectly affect ambrosia beetle attacks due to a moderation of tree stress signals used by the beetle to locate suitable hosts [67]. In the present study, the pre-treatment with azoxystrobin reduced the mean brood size produced by *X. compactus* foundresses (Figs 1 and 2). On the other hand, a preventive fungicide application with azoxystrobin had variable effectiveness in reducing *X. germanus* attacks, but significantly reduced the number of galleries with fungal growth on flooded treated *Cercis canadensis* L. trees [31].

The present study also demonstrates how azoxystrobin application can significantly reduce the lesion length due to wood colonization by the fungal mutualist, potentially related to the active ingredient systemic activity. Moreover, this broad-spectrum fungicide can have a direct action towards fungi or could lead to plant physiological changes, such as promotion of plant growth and stress tolerance and decrease of ethylene biosynthesis [73–75]. The laboratory trials with azoxystrobin showed inhibition of the mycelial growth of the *X. germanus* fungal mutualist [76]. Preventative and curative treatments with pyraclostrobin + fluxapyroxad reduced ambrosia beetle attacks on treated flowering dogwoods *Cornus florida* L. [35]. Addesso et al. (2018) [67] reported the suitability of pyraclostrobin + boscalid to reduce ambrosia beetle attacks and Phytophthora root rot disease severity in flooded plants. The same mixture reduced attacks post-landing of ambrosia beetle on *Magnolia* sp. trees seven days before flood stress, suggesting a possible short-range toxicity or avoidance. Moreover, trifloxystrobin + triadimefon reduced attacks to ethanol-injected trees by pre-landing effects suggesting a possible longer-range repellence [34].

We observed no or low efficacy to control *X. compactus* infestations when testing the novel triazole mefentrifuconazole. A reduction in the beetle mean brood size was observed only with pre-treatments with mefentrifuconazole by soil applications at 1 DAT and 3 DAT. The potential of triazoles to manage ambrosia beetles has been evaluated in some previous studies. In particular, tebuconazole and propiconazole + emamectin benzoate were found to be effective in *Euwallacea* spp. nr. *fornicatus* (Eichhoff) colonisation attempts on California sycamore (*Platanus racemosa* Nutt.) [36,37]. In addition, the efficacy of tebuconazole and propiconazole in inhibiting fungal growth of a fungus associated to *X. compactus* has been investigated through *in vitro* trials [38,39]. Propiconazole was found to be effective *in vitro* against the laurel wilt pathogen *Raffaelea lauricola* (Harr., Fraedrich & Aghayeva), the fungal symbiont of *Xyleborus glabratus* (Eichhoff) [77]. Roberts et al. (2024) [68] proved that propiconazole reduced colony establishment success of *E. fornicatus* and the lesion lengths caused by its mutualist *Fusarium euwallaceaea* (Freeman, Mendel, Aoki & O’Donnel) on American sweetgum (*Liquidambar styraciflua* L.). However, mefentrifuconazole did not reduce fungal lesion length in extended laboratory bioassays we conducted with *X. compactus* and its mutualist *A. xylebori*.

Biocontrol agents perform their function through various actions, including mycoparasitism, induced plant resistance, competition for nutrients and space, antibiotic challenge or plant growth promotion. In particular*, Trichoderma* spp. are potential root colonisers and provide benefits to their hosts by inducing changes at the biochemical and physiological level, Systemic Acquired Resistance (SAR) and ISR and plant growth as Plant Growth Promoting Fungi (PGPF), while *Bacillus* spp. include Plant Growth Promoting Rhizobacteria (PGPR) which assist the plant in nutrient uptake and root colonisation, stimulate plant development and are elicitors of ISR [78–82]. However, few studies focused on the use of these microbial agents to disrupt interactions between ambrosia beetle and their mutualistic fungi in the framework of developing novel management approaches.

The potential of *Trichoderma* spp. and *Bacillus* spp. to manage *X. compactus* infestations was recently investigated in the laboratory by Gugliuzzo et al. (2022) [25], who tested several biocontrol agents, including *T. asperellum* strain T34, *T. asperellum* strain ICC 012 + *T. gamsii* strain ICC 080 and *B. amyloliquefaciens* strain D747. According to their results, all these biocontrol agents significantly suppressed the growth of the fungal mutualist *A. xylebori* both *in vitro* and *in vivo* bioassays. Similarly, the growth of *Ambrosiella roeperi* (Harr. & McNew) (fungal mutualist of *X. crassiusculus*) and *Ambrosiella grosmanniae* (McNew, Mayers & Harr.) (fungal mutualist of *X. germanus*) was reduced by *T. afroharzianum* (formerly *T. harzianum*) strain T-22 and *T. asperellum* or *T. atroviride*, respectively, *in vitro* assays [42,83]. Subsequently, *Trichoderma* spp. significantly reduced brood production on *X. crassiusculus* and *X. germanus* in complementary laboratory bioassays, after exposure of foundresses to treated beech stems [42]. The use of *T. harzianum* strain T-22 + *T. virens* strain G-41 was evaluated for the control of Phytophthora root rot and ambrosia beetles on *C. florida* after flood events, showing a weak reduction in beetle attacks on treated trees [35]. A field trial with *Bacillus amyloliquefaciens* (formerly *B. subtilis*) QST 713 on California sycamore trees also showed no significant reduction in *Euwallacea* spp. attacks more than one month after treatment [33].

Results from our extended laboratory bioassays showed that none of the microbial-based fungicides, applied to the soil, reduced the proportion of beetle establishing galleries, the growth of the mutualist fungus and the beetles brood size. However, the *Trichoderma asperellum* strain T34 significantly reduced the lesion length of infested bay laurel plants. Both PGPF and PGPR can induce plant resistance not only towards soil-borne pathogens, but also to aerial or vascular pathogens in distant plant parts, either in cultivated crops or in forest environment [57,62,84,85]. Our results suggest that root colonisation by *T. asperellum* strain T34 may have induced systemic plant resistance towards *A. xylebori*. A similar result was obtained by testing PGPR to control Ophiostomatoid fungi, commonly associated to wood-boring beetles. Specifically, *in vitro* tests confirmed the potential antibiosis activity of different strains of *Bacillus velezensis*, *Paenibacillus peoniae* strain AP294 and *B. altitudinis* strain AB69, which inhibited the growth of *Leptographium terebrantis* (Barras & Perry) and *Grosmannia huntii (*Rob & Jeffr), agents of wood blue stain and root disease of *Pinus* species. In addition, *B. pumilus* strains INR7 and SE-34 and *Serratia marcescens* strain 90–166 have induced ISR in *Pinus taeda* L. seedlings after being introduced in the root ball and having inoculated plants with fungal pathogens along the stems. The ISR was proven by the significant reduction of lesions and occlusion length caused by Ophiostomatoid fungi [62]. The same *in vivo* tested BCAs have been reported to cause ISR on conifers towards *Cronartium quercium* (Berk.) Miyabe ex Shirai f.sp. *fusiforme,* responsible for the fusiform rust disease in pine stem and branches [61].

Root colonisation by the other tested microbials, i.e., *T. asperellum* strain ICC 012 + *T. gamsii* strain ICC 080 and *B. amyloliquefaciens* D747 may not have induced any change in the beetle-fungus mutualistic interaction, as potentially occurred with *T. asperellum* T34. Further molecular studies will be required to determine how the soil application of specific microbial BCAs could affect this complex plant-beetle-fungus interaction, e.g., induced plant resistance, plant growth promotion, etc.

Some fungicides may have repellent properties, as observed also for certain ambrosia beetles [34]. However, the potential of fungicide applications to repel *X. compactus* requires further study. Evidence of repellence toward ambrosia beetle species has been reported when testing other natural substances, such as for example plant essential oils (i.e., *Rosmarinus officinalis* L. cv. *verbenoniferum* and *Carlina acaulis* L.) [40]. In the present study, laboratory choice bioassays did not show any preference of *X. compactus* between fungicide-treated and untreated bay laurel stems. However, other fungicides may have repellent properties, as well as other natural substances including botanicals and microbial BCAs, which should be further tested when developing management strategies aimed at manipulating insect behaviour.

## Conclusion

Overall, this study is the first one providing baseline data and new insights for the use of fungicides as potential tools to indirectly affect the infestation success of the ambrosia beetle *X. compactus* through the suppression of its nutritional mutualist, *A. xylebori.* We demonstrate that some fungicides may be considered candidates to be included into IPM programs aiming at managing this invasive ambrosia beetle. However, fungicide performance can be directly dependent on the application timing and methodology, i.e., soil or spray. Consequently, future field trials must be developed in a context-dependent way in order to identify the optimal application methodology. These studies should strictly consider several aspects of treatment recommendations, including: mode of action of each active ingredient, application timing, host plant, beetle phenology, and specific environment, e.g., nurseries, orchards, urban areas, etc.

## Supporting information

S1 Fig a) Flooded bay laurel plant with detail of the pot-in-pot system; b) release of a beetle female inside a vial appositely attached along the main plant stem; c) evidence of sawdust accumulation resulting from the boring activity of the released beetle female; d) assessment of the vascular lesion length.
(DOCX)
