# Supplementary material for: Exploring the potential of synthetic and biological fungicides for managing the fungus-farming ambrosia beetle Xylosandrus compactus
Source: PLoS One. 2025 Jul 31;20(7):e0329063. doi: 10.1371/journal.pone.0329063 (PMC12312887; doi:10.1371/journal.pone.0329063)

**Supporting Information Figure S1.**

a) Flooded bay laurel plant with detail of the pot-in-pot system; b) release of a beetle female inside a vial appositely attached along the main plant stem; c) evidence of sawdust accumulation resulting from the boring activity of the released beetle female; d) assessment of the vascular lesion length.


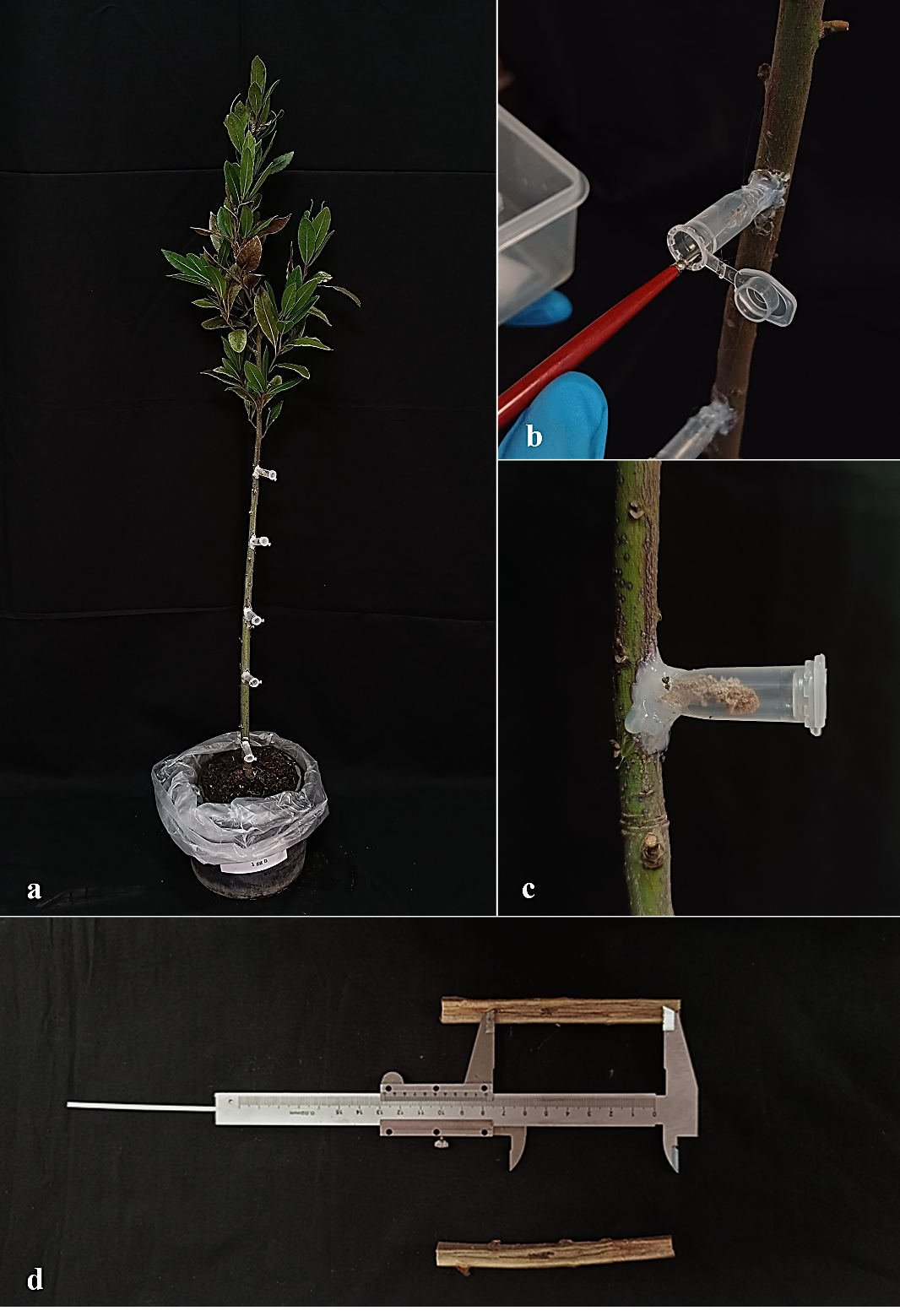

Supplement: S1 Fig — a) Flooded bay laurel plant with detail of the pot-in-pot system; b) release of a beetle female inside a vial appositely attached along the main plant stem; c) evidence of sawdust accumulation resulting from the boring activity of the released beetle female; d) assessment of the vascular lesion length. (DOCX) [file pone.0329063.s001.docx]
